# Supplementary material for: Exosomes from EGFR-Mutated Adenocarcinoma Induce a Hybrid EMT and MMP9-Dependant Tumor Invasion
Source: Cancers (Basel). 2022 Aug 3;14(15):3776. doi: 10.3390/cancers14153776 (PMC9367273; doi:10.3390/cancers14153776)
Supplement: Supplementary file 1 [file cancers-14-03776-s001.zip › cancers-1814931-supplementary.pdf]

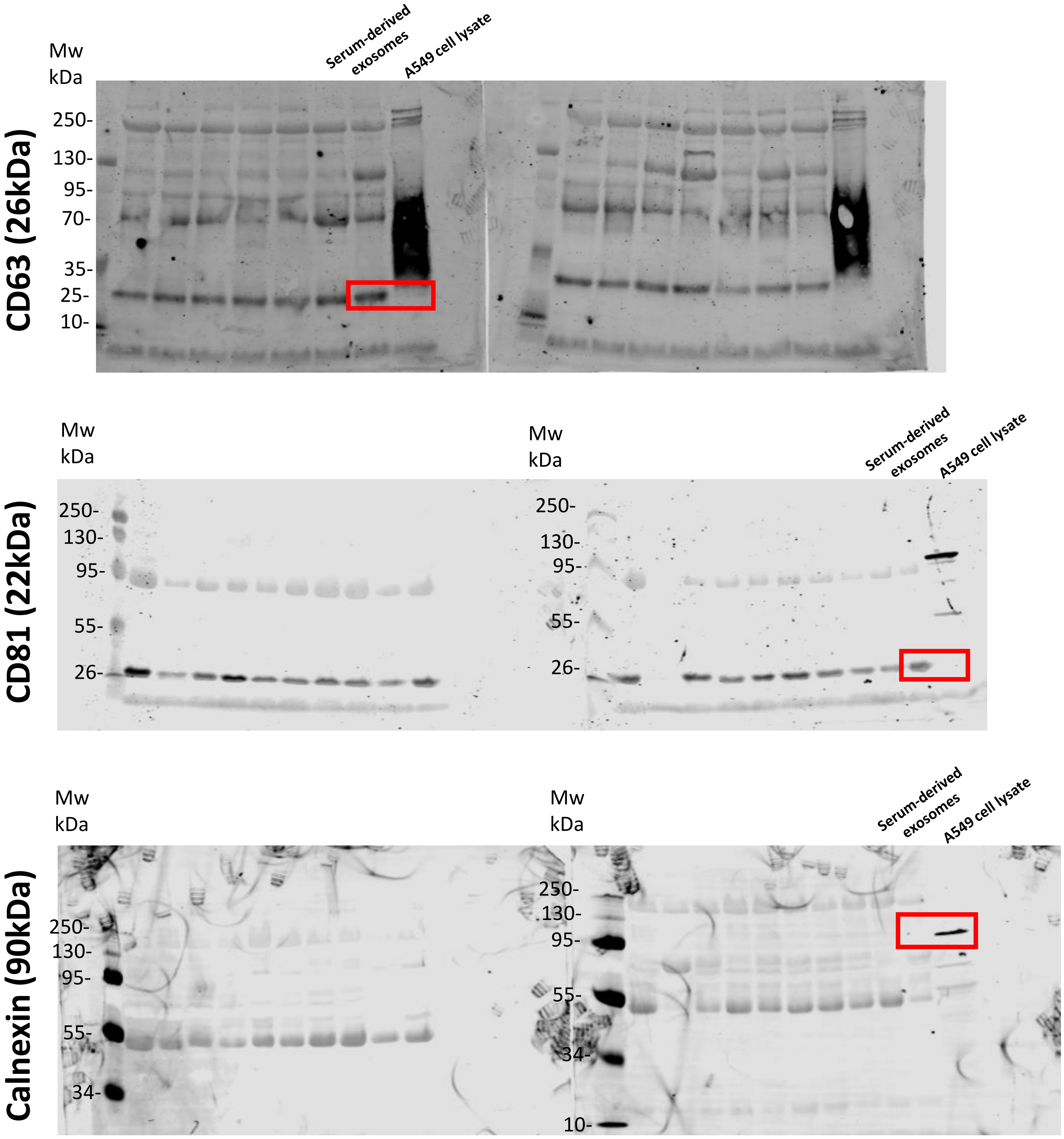

Figure S1: Original whole Western-blot Images for Figure 1C. The densitometry reding for the western-blot could be found in supplementary Table 2.

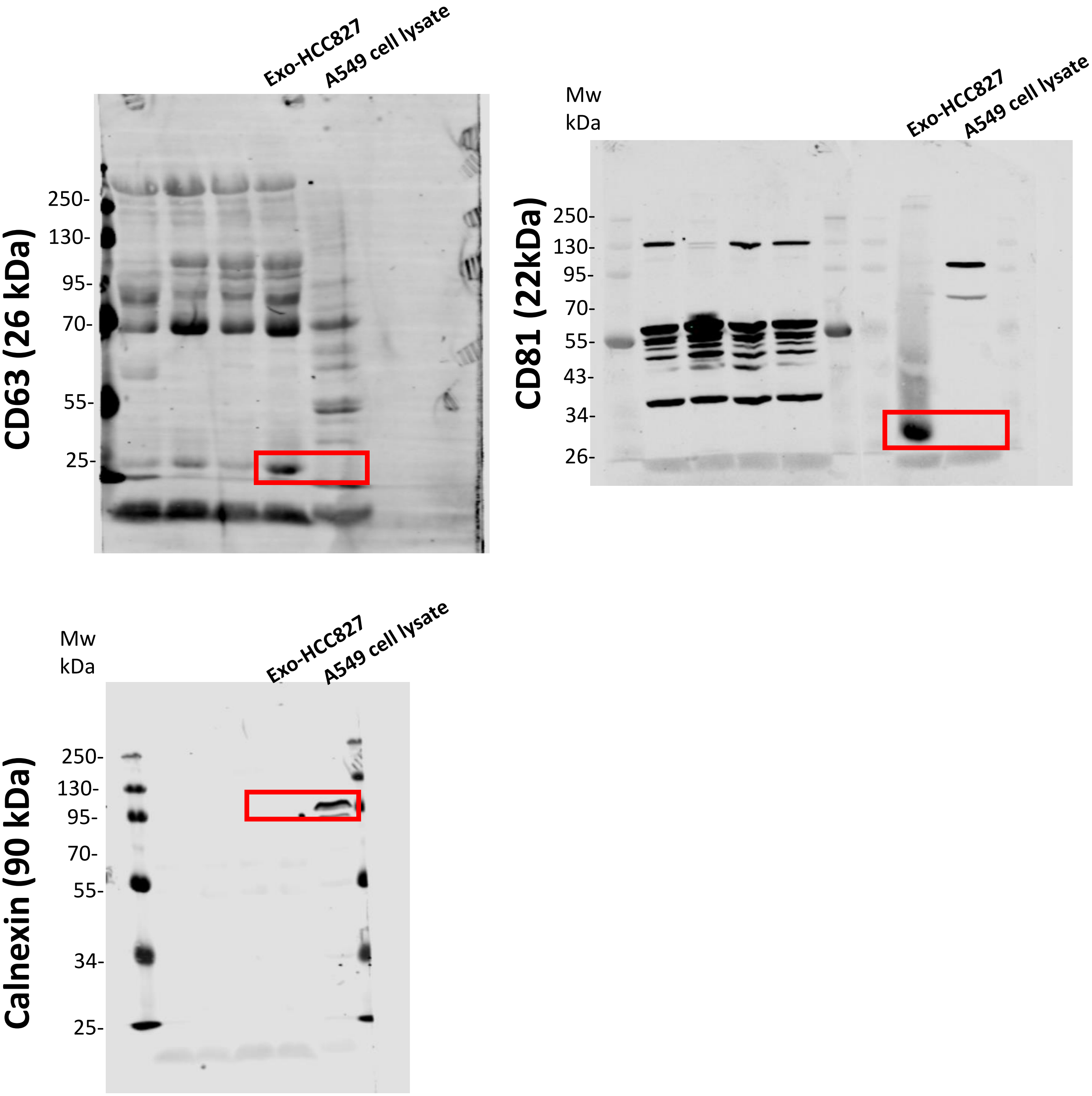

Figure S2: Original whole Western-blot Images for Figure 2C. The densitometry reding for the western-blot could be found in supplementary Table 3.

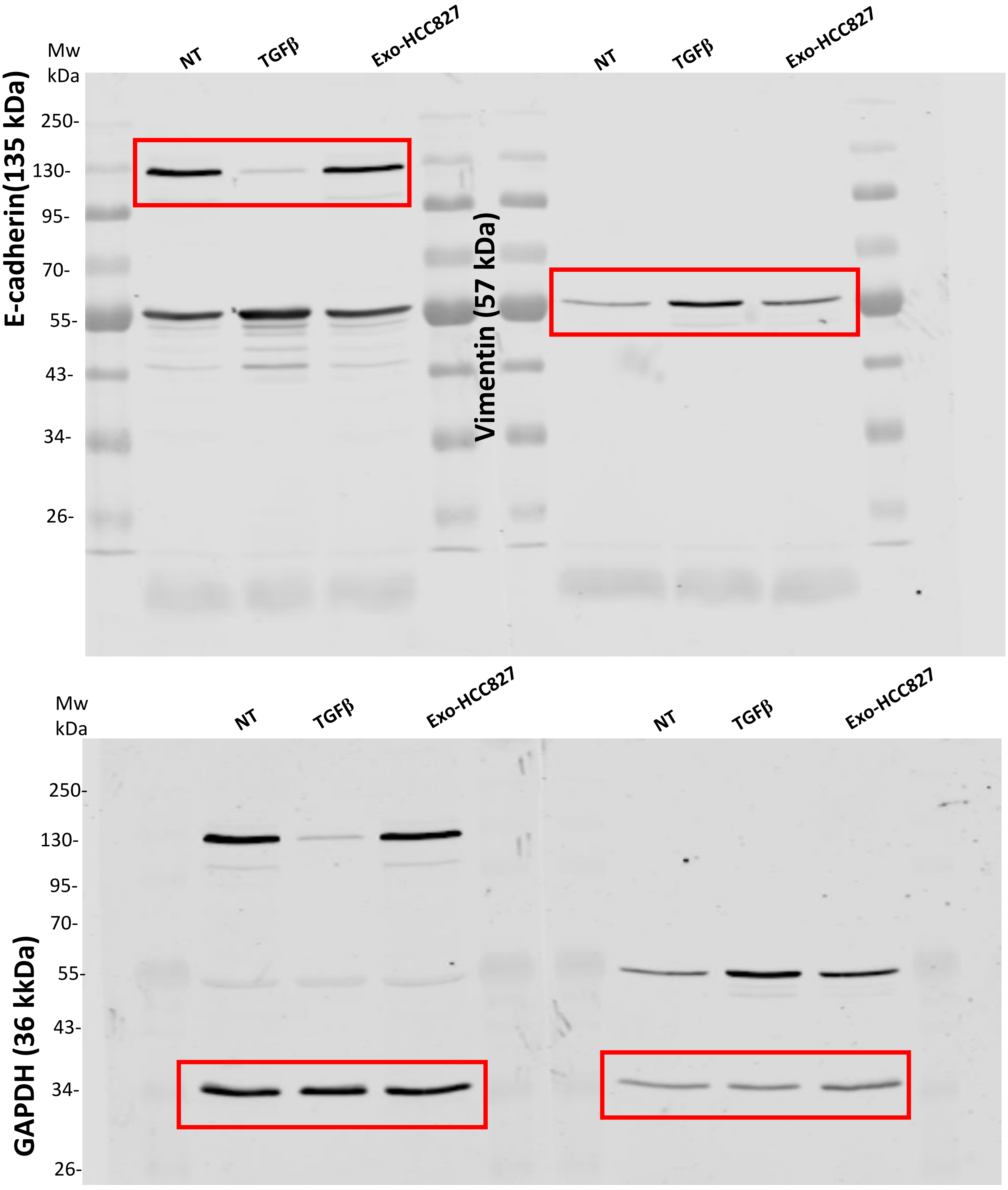

Figure S3: Original whole Western-blot Images for Figure 4B. The densitometry reding for the western-blot could be found in supplementary Table 4.

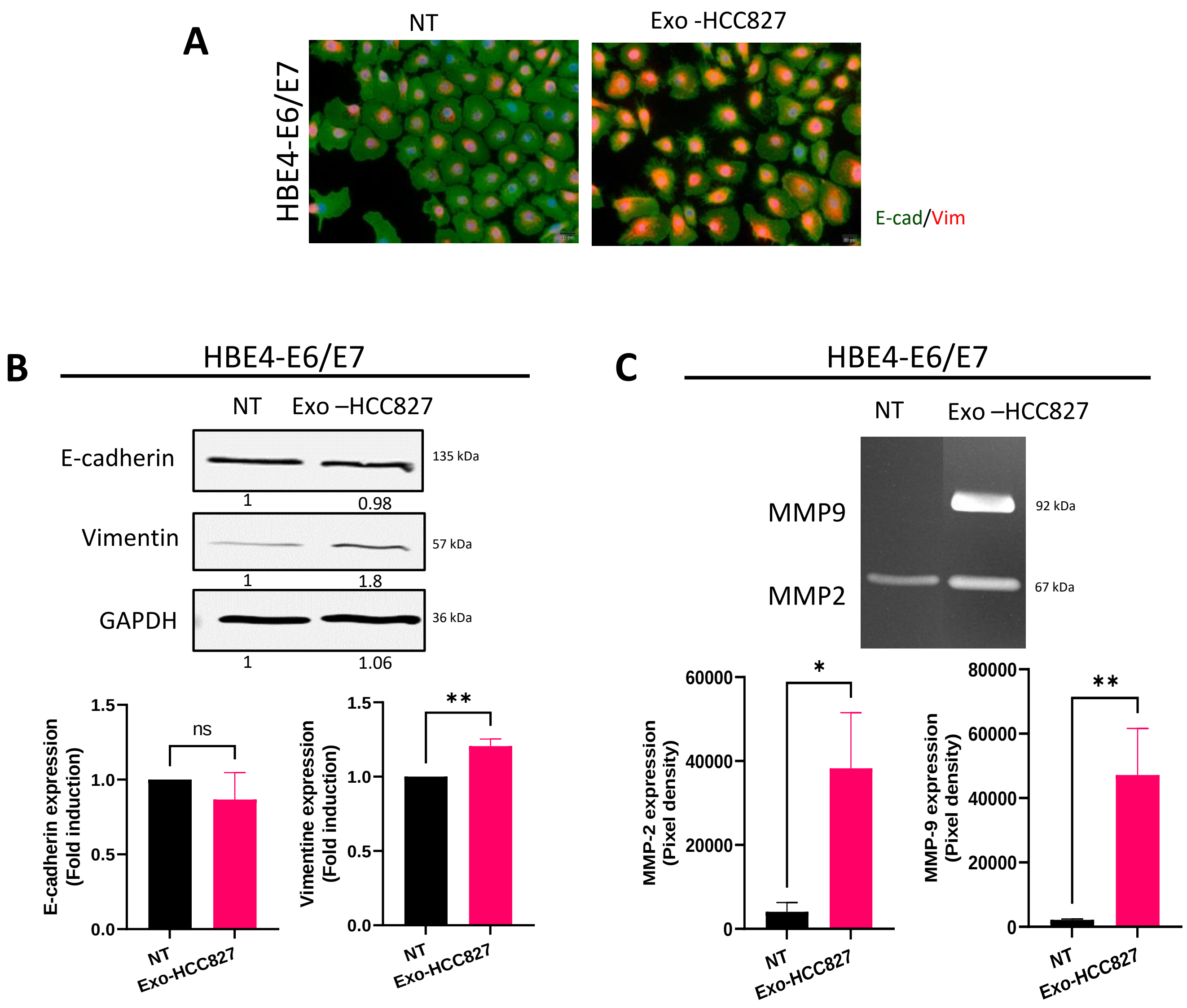

Figure S4: Exosomes derived from HCC827 cell line promote extracellular matrix degradation and EMT **A)** Immunofluorescence detection of Vimentin (red) and E-cadherin (green) in HBE4 E6/E7 cells treated with exosomes isolated from HCC827 supernatant. Nuclei were stained with DAPI (blue). Scale bar = 20 μm. **B)** Western blot analysis and quantification of Vimentin and E-cadherin expression in HBE4 E6/E7 cells treated with exosomes isolated from HCC827 supernatant. GAPDH is used as a loading control. **C)** Zymography analysis and quantification of HBE4 E6/E7 cells treated with exosomes isolated from HCC827 supernatant.

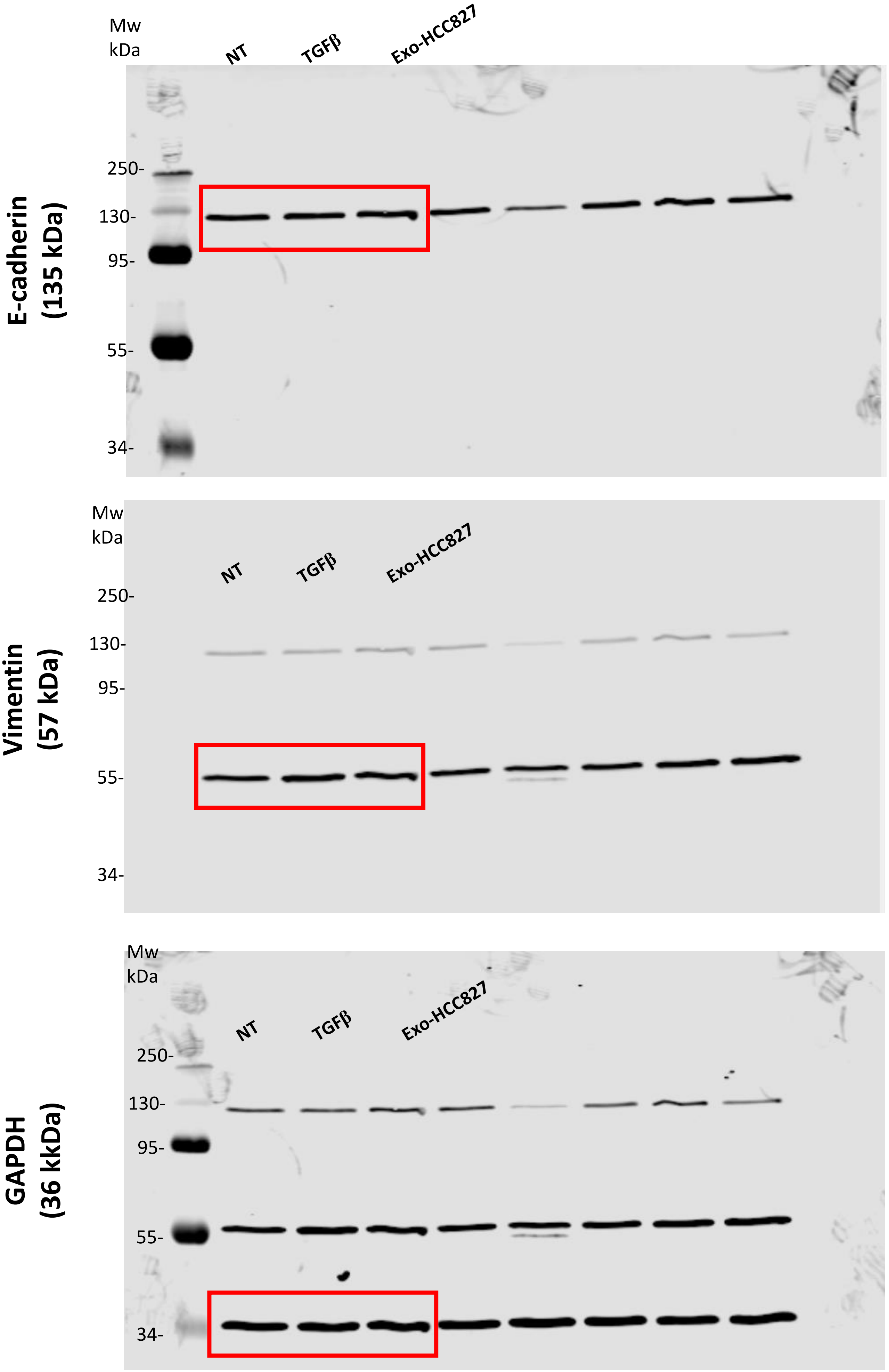

Figure S5: Original whole Western-blot Images for Supplementary Figure 4. The densitometry reding for the western-blot could be found in supplementary Table 5.

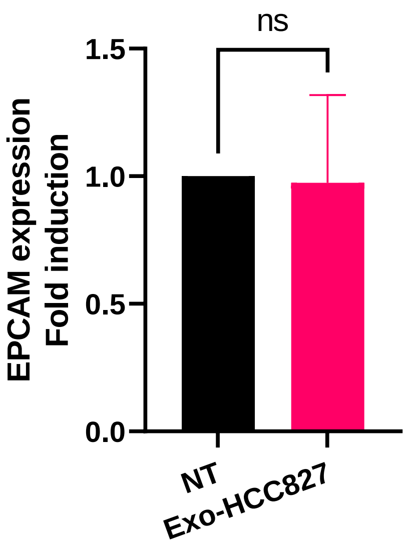

Figure S6: Exosomes from HCC827 cell line don’t alter tight junction expression. Quantification analysis of EPCAM expression obtain by RT-qPCR of protein extracts of A549 cells treated with exosomes isolated from HCC827 supernatant.

Table S1: Patient characteristics

Age	
Mean	69 (50-86)
Gender	
Female	9
Male	7
Smoker (yes/no/Ex-smoker)	6 / 5 / 9
Clinical stage	
I	5
II	3
III	5
IV	2
Squamous cell lung Cancer	5
Lung adenocarcinoma	10
wild type	5
EGFR mutation	5

Table S2: Densitometry reading for Figure 1C

Target	Sample ID	Pixel density
CD63	Serum-derived exosomes	28664
	A549 whole cell lysate	2852
CD81	Serum-derived exosomes	23056
	A549 whole cell lysate	2341
Calnexin	Serum-derived exosomes	744
	A549 whole cell lysate	28942

Table S3: Densitometry reading for Figure 2C

Target	Sample ID	Pixel density
CD63	Exo-HCC827	52516
	A549 whole cell lysate	2104
CD81	Exo-HCC827	56496
	A549 whole cell lysate	645
Calnexin	Exo-HCC827	69
	A549 whole cell lysate	74946

Table S4: Densitometry reading for Figure 4B

Target	Sample ID	Pixel density	Relative expression
E-cadherin	A549 Non-treated	48579	1.05
	A549 exposed to Exo-HCC827	45919	0.97
Vimentin	A549 Non-treated	15349	0.45
	A549 exposed to Exo-HCC827	33729	0.71
GAPDH (for E-cadherin)	A549 Non-treated	46019	
	A549 exposed to Exo-HCC827	46989	
GAPDH (for vimentin)	A549 Non-treated	33807	
	A549 exposed to Exo-HCC827	47014	

Table S5: Densitometry reading for Figure S4

Target	Sample ID	Pixel density	Relative expression
E-cadherin	HBE4-E6/E7 Non-treated	34995	0.72
	HBE4-E6/E7 exposed to Exo-HCC827	34864	0.71
Vimentin	HBE4-E6/E7 Non-treated	8023	0.16
	HBE4-E6/E7 exposed to Exo-HCC827	14547	0.29
GAPDH	HBE4-E6/E7 Non-treated	48153	
	HBE4-E6/E7 exposed to Exo-HCC827	48987	

Video S1

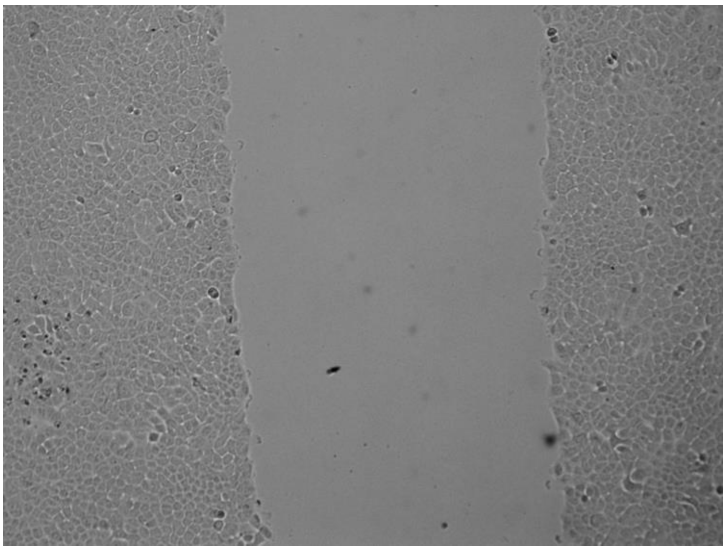

NT

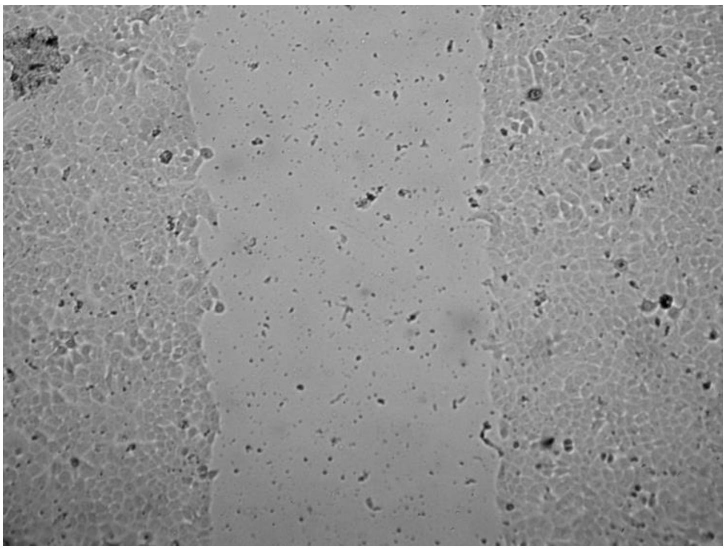

Exo –HCC827

Video S1: Exosomes from HCC827 cell line increase wound healing
Video of scratch inflected to A549 cell monolayers treated with exosomes isolated from HCC827 supernatant.(48h)
